# Late Recognition of Autism Previously Diagnosed as Schizophrenia Following a Brief Psychotic Episode: A Case Report

**DOI:** 10.7759/cureus.106337

**Published:** 2026-04-02

**Authors:** Alfredo de Jesús Garza Guerra, Elisa del Rocio Mata Cortes, Gabriela Hilian Adame Rocha, Ana Cecilia Montemayor Mancias

**Affiliations:** 1 Psychiatry, Hospital Universitario Dr. José Eleuterio González, Nuevo Leon, MEX; 2 Psychiatry, Jalisco Institute of Mental Health and Addictions, University of Guadalajara, Jalisco, MEX

**Keywords:** autism, late diagnosis, longitudinal assessment, misdiagnosis, neurodevelopment, psychotic episode, schizophrenia spectrum

## Abstract

Autism is a neurodevelopmental condition characterized by differences in social communication and patterns of behavior, interests, or activities. Many individuals remain unrecognized until adulthood or later life, particularly those who develop adaptive strategies that allow them to navigate social and environmental demands. This may complicate clinical interpretation when psychiatric symptoms arise. We present the case of a 74-year-old individual whose first contact with mental health services occurred at age 23 following a brief psychotic episode, leading to a long-standing diagnosis within the schizophrenia spectrum. Over subsequent decades, there was no recurrence of psychotic experiences or evidence of progressive functional decline. A later longitudinal reassessment, informed by developmental history, collateral information, and standardized instruments, identified a pattern of traits consistent with previously unrecognized autism. Antipsychotic medication was gradually discontinued without adverse clinical outcomes. This case highlights the importance of integrating developmental trajectories and longitudinal course into psychiatric evaluation, particularly in individuals with well-established adaptive functioning. It also illustrates how enduring neurodevelopmental differences may be misinterpreted as chronic psychiatric conditions when assessment relies predominantly on cross-sectional symptomatology.

## Introduction

Autism is a neurodevelopmental condition as defined in Diagnostic and Statistical Manual of Mental Disorders, Fifth Edition (DSM-5) by persistent differences in social communication and interaction across multiple contexts, together with patterns of behavior, interests, or activities that may be more intense, focused, or repetitive, present from early developmental stages, even if they become more evident later in life [1,2]. These characteristics are part of neurobiological diversity and can manifest heterogeneously across the lifespan [1,2]. In recent decades, there has been increasing recognition that a significant number of autistic individuals reach adulthood and even older age without being identified previously, particularly those who develop adaptive strategies over time [3-6]. This under-recognition is associated with historical shifts in conceptual frameworks, biases in recognition processes, and the frequent co-occurrence of mental health conditions that may influence clinical presentation [7-10].

Schizophrenia is a severe mental disorder characterized by disturbances in perception, thought, and behavior, including positive symptoms such as hallucinations and delusions, as well as negative symptoms involving reduced emotional expressivity, diminished motivation, and social withdrawal. Cognitive and disorganization symptoms are also commonly present and contribute to functional impairment [1,11].

Although autism was historically conceptualized within early formulations of childhood schizophrenia, contemporary frameworks clearly distinguish autism as a neurodevelopmental condition from schizophrenia spectrum disorders. Despite this distinction, some phenomenological overlap may occur, particularly in domains such as social communication, emotional expressivity, and behavioral patterns, which may resemble negative symptoms [11-13]. Additionally, autistic individuals may show increased vulnerability to psychotic experiences across the lifespan, with reported rates ranging from approximately 10% to 35%, while the prevalence of diagnosable psychotic disorders remains considerably lower, typically estimated at around 3-6% [14,15]. Within this framework, one of the main challenges in recognition lies in a bias toward cross-sectional evaluations focused on the current clinical state, guided by observable manifestations at a given time, without adequately integrating developmental history or longitudinal trajectory. As a result, persistent characteristics may be misinterpreted as manifestations of an ongoing clinical episode rather than as part of a broader and stable pattern across the lifespan. Consequently, failure to recognize autism across the lifespan may lead to acute psychotic episodes being interpreted within categorical frameworks such as schizophrenia, without considering an underlying neurodevelopmental condition, with important implications for clinical understanding, treatment decisions, and the individual’s subjective experience [9,11,14].

In this context, we present the case of an individual whose autism was not recognized until older age and whose first contact with mental health services occurred following a brief psychotic episode that was initially interpreted within the schizophrenia spectrum. A longitudinal reassessment integrating developmental history led to the identification of autism-related characteristics that had gone unnoticed for decades.

## Case presentation

We present the case of a 74-year-old male with no history of mental health care during childhood or adolescence, whose first contact with psychiatric services occurred at age 23 following an acute-onset psychotic episode. He had no relevant medical or psychiatric history at the time of evaluation, reporting only occasional, moderate alcohol use.

Premorbid functioning was characterized by longstanding differences in social communication and behavior present since childhood. The individual showed a preference for solitary activities, with limited interest in peer interactions and difficulty engaging in reciprocal social communication. His communication style was direct, with reduced use of nonverbal cues and limited spontaneous social engagement. Emotional expressivity was described as reduced but stable across contexts. He also demonstrated a strong preference for routines and predictable environments, along with focused interests from an early age. Academic history was unremarkable, with completion of secondary education comparable to that of his siblings, without reported learning difficulties.

According to information provided by the individual and family members, physical and neurological examinations, as well as laboratory and radiological investigations conducted at the time of the initial presentation, were within normal limits, with no significant abnormalities identified.

At age 23, the clinical presentation included predominantly auditory hallucinations, described as voices commenting on the individual’s actions, occurring several times per day during the acute phase. These symptoms emerged in the context of a significant life stressor, namely the death of the individual’s father, with onset occurring within weeks of this event. The experiences were intrusive and distressing, contributing to heightened anxiety and behavioral disorganization. Delusional ideas were also present, mainly involving persecutory themes. The overall intensity of psychotic symptoms was moderate to severe, with positive symptoms lasting less than one month.

Treatment with perphenazine was initiated, with subsequent resolution of psychotic symptoms within the same time frame. During follow-up, features such as reduced eye contact, a direct communication style with limited use of implicit or nonverbal cues, and lower emotional expressivity were observed. These characteristics, present since early developmental stages and stable over time, were initially interpreted as residual or negative symptoms, leading to a diagnosis within the schizophrenia spectrum. The individual subsequently participated in supportive group-based interventions for individuals with psychotic disorders on a monthly basis, demonstrating appropriate engagement and being described as reflective and analytical, with no behavioral disturbances.

Over subsequent decades, no recurrence of psychotic symptoms or evidence of progressive functional decline was documented. The individual remained in treatment under relapse prevention recommendations, although he consistently expressed that he did not identify with the diagnosis.

From a longitudinal perspective, a stable pattern of functioning was observed. The individual began working at approximately 20 years of age and maintained autonomy, engaging in seasonal work involving the preparation and sale of desserts. At age 74, he remained engaged in work activities by personal choice, despite having adequate family support. A persistent and highly focused interest in plant care was noted as a central aspect of daily life.

Consistent characteristics included a preference for structured routines and predictable daily activities. Sensory differences were also present, including tactile hypersensitivity, with a preference for wearing layered, long-sleeved clothing regardless of weather conditions as a means of sensory regulation. This behavior had previously been interpreted as disorganized. The individual reported no interest in romantic relationships and had never been married, but maintained close family relationships and was well integrated socially. Social functioning remained stable over time, with no significant changes between the period of the acute psychotic episode and later life.

The individual lives independently and maintains functional autonomy in daily activities, with close support from siblings and other family members.

He was followed by our team from his early 70s onward in the psychiatry department. Physical and neurological examinations, vital signs, and laboratory and radiological investigations, including brain magnetic resonance imaging, were unremarkable. A mental status examination at the time of reassessment showed that the individual was alert and oriented, with no evidence of hallucinations, delusions, or disorganized thought processes. Affect was stable, and communication style remained consistent with previously described patterns.

A comprehensive reassessment was conducted by a multidisciplinary team, including psychiatrists with experience in adult neurodevelopmental conditions, incorporating detailed developmental history and collateral information from family members. The evaluation was based on DSM-5 criteria and supported by standardized instruments, including the Autism-Spectrum Quotient (AQ) and the Ritvo Autism Asperger Diagnostic Scale-Revised (RAADS-R). The individual scored 38 on the AQ and 152 on the RAADS-R, both within ranges consistent with autism.

At the time of presentation at age 74, the individual was receiving perphenazine at a maintenance dose of 8 mg daily, administered at night. Antipsychotic medication was gradually discontinued over approximately one year under clinical supervision, with no adverse effects or recurrence of psychotic symptoms.

At the most recent follow-up, the individual remains clinically stable, with preserved functional autonomy and quality of life. He continues under regular outpatient follow-up on a quarterly basis, with no pharmacological treatment currently prescribed. Supportive psychotherapy is ongoing.

Integration of longitudinal information revealed a pattern consistent with previously unrecognized autism. Family history suggested a broader neurodevelopmental context, including two nieces diagnosed with attention-deficit/hyperactivity disorder (ADHD) and broader autism phenotype traits in other family members.

Following the diagnosis, guidance was provided to the individual and family, including psychoeducation regarding the neurodevelopmental nature of autism and recommendations to maintain structured routines and support adaptive strategies in daily life.

## Discussion

This case describes an individual whose autism remained unrecognized throughout life and who first came into contact with mental health services during early adulthood following a brief psychotic episode. The manifestations observed at that time were initially interpreted within categorical frameworks associated with schizophrenia. However, the integration of developmental history and longitudinal trajectory later allowed for the recognition of a pattern consistent with autism, present since early stages of life and previously unrecognized. This contrast highlights the importance of considering the full life course in mental health evaluation [4-6].

This case also illustrates how misdiagnosis may arise from reliance on cross-sectional assessment, limited integration of developmental history, and phenomenological overlap between autistic characteristics and negative symptoms. Differences in social communication, reduced emotional expressivity, and preference for routines may be interpreted as indicators of a chronic psychotic condition when their developmental continuity is not considered, particularly in the context of an acute psychotic episode [11,12,14].

It further draws attention to the subjective and social implications of early diagnostic interpretations that may not be experienced as representative by the individual. Such interpretations can carry significant weight within healthcare systems and influence how personal narratives and lived experiences are validated or dismissed over time [16-18].

Recognition of autism in later life has increased in recent years, reflecting not necessarily a change in prevalence but an expansion in how autism is understood across the lifespan [1,2]. Many individuals who grew up in contexts with more restrictive diagnostic frameworks were not identified, particularly those who developed adaptive strategies or maintained functional stability in structured environments [4-6].

Late recognition does not imply a recent emergence of characteristics but rather a shift in their interpretation. It has been suggested that up to nine in 10 autistic individuals over the age of 50 may remain unrecognized or may have been previously interpreted within other diagnostic frameworks [19].

One of the central challenges in such cases lies in distinguishing between longstanding autistic characteristics and what, within schizophrenia spectrum frameworks, would be described as negative symptoms. This distinction depends not only on observable features but also on their developmental trajectory and underlying organization. Similar features, such as reduced emotional expressivity, differences in social interaction, or diminished verbal output, may arise from distinct mechanisms across conditions, complicating clinical interpretation [11-13].

Following a psychotic episode, characteristics such as reduced emotional expressivity, differences in communication style, structured routines, and focused interests may be interpreted as residual symptoms. However, in some individuals, these represent stable patterns present since early developmental stages. Without a longitudinal perspective, there is a risk of attributing these patterns to an ongoing psychotic process, reinforcing assumptions of chronicity and favoring a schizophrenia spectrum diagnosis [12,14].

Several studies suggest that autistic individuals may have increased vulnerability to psychotic experiences across the lifespan [12,15]. These experiences are often limited in duration and closely related to contextual factors, such as environmental changes, increased demands, or significant stress. In this sense, they may be better understood as context-dependent phenomena rather than indicators of a persistent condition. This distinction is essential for differentiating such presentations from chronic psychotic disorders, particularly in the absence of recurrence or functional decline [12,15].

This perspective aligns with dimensional models of psychosis, in which psychotic experiences are conceptualized along a continuum rather than as discrete categories [12].

From a clinical perspective, this case underscores the importance of systematically incorporating developmental history and longitudinal trajectory into adult psychiatric evaluation. It also highlights the need to revisit initial diagnostic interpretations, particularly when clinical evolution does not follow expected patterns. Considering previously unrecognized autism may support a more accurate understanding of the individual’s experience and guide more context-sensitive care.

This includes not only therapeutic decision-making, such as avoiding prolonged use of antipsychotic medication in the absence of ongoing psychotic phenomena, but also how experiences are communicated and understood at both personal and social levels. Diagnostic interpretations are not neutral; they shape self-perception, expectations of chronicity, and access to social and occupational opportunities [11,17].

Future research should focus on longitudinal studies integrating developmental history, life course trajectories, and contextual factors to improve diagnostic differentiation between autism and psychotic disorders. Further work is also needed to better understand the phenomenological overlap between autistic characteristics and negative symptoms, particularly in adult and late-life populations, as well as the clinical course of autistic individuals who experience brief psychotic episodes.

Informed consent was obtained from the individual for publication of this case report.

To support clinical differentiation, Table 1 summarizes key features distinguishing autism, brief psychotic disorder, and schizophrenia spectrum disorders across longitudinal, phenomenological, and functional domains.

**Table 1:** Clinical differentiation between autism, brief psychotic disorder, and schizophrenia spectrum disorders The features presented are intended as general clinical patterns and may vary across individuals; accurate differentiation requires integration of developmental history and longitudinal course.

Domain	Autism (longitudinal trajectory)	Brief psychotic disorder	Schizophrenia spectrum disorders
Onset	Early developmental onset [2,3,7]	Acute onset, often in response to stress [1]	Typically late adolescence or early adulthood [1,12]
Course	Stable over time (persistent traits) [2,3]	Episodic, duration <1 month with full remission [1]	Chronic or episodic with progressive deterioration [1,12]
Psychotic symptoms	May occur, usually brief, stress-related, without sustained delusional structure [12,14]	Present during the episode, with full resolution [1]	Persistent or recurrent (delusions, hallucinations, disorganization) [1,12,14]
Subclinical psychotic experiences	More frequent; transient, stress-related [12,14]	Not characteristic outside the episode	May precede onset (prodromal phase) [12]
Affect	Atypical but consistent emotional expression [11,13]	Affective lability during the episode [1]	Progressive affective flattening [11,13]
Social communication	Lifelong structural differences in reciprocity, pragmatics, and nonverbal communication; reduced or atypical eye contact; preference for structured interactions [2,3]	Transient impairment during episode [1]	Secondary deterioration due to illness [2,3,12]
Interests	Intense, specific, and stable; may play a central role in daily life [2,3]	Not characteristic	May become reduced or disorganized [12]
Routines / rigidity	Preference for predictability; distress with change [2]	Not characteristic	Not a core feature (may show cognitive rigidity) [12]
Sensory profile	Sensory differences (e.g., hypersensitivity to tactile, auditory, or visual stimuli); use of strategies for sensory regulation [2,3]	Not characteristic	Not typical
Insight	Reflective insight [14]	Typically restored after episode [1]	Often limited or absent [1,14]
Global functioning	Relatively stable, often with high adaptive cost [4,6,10]	Full return to baseline functioning [1]	Frequent progressive functional decline [4,6,10]
Comorbidity and stress response	High psychiatric comorbidity; stress-related dysregulation [9,10,17]	Usually linked to acute stressors [1]	High comorbidity with functional impairment [9]
Response to antipsychotics	Limited in the absence of structured psychosis [12,14]	Good response, usually short-term [1]	Generally required for symptom control [1,14]

## Conclusions

This case illustrates how the absence of a developmental and longitudinal perspective in psychiatric evaluation may lead to enduring misinterpretations of neurodevelopmental characteristics as chronic psychiatric conditions. It highlights the importance of adopting a lifespan-oriented and context-sensitive approach that allows for the revision of diagnostic formulations over time. Recognizing autism in later life not only contributes to a more accurate clinical understanding but also supports more individualized and meaningful care.

More broadly, this case underscores the need for flexible and integrative diagnostic frameworks that move beyond strictly categorical models, acknowledging the complexity, heterogeneity, and developmental continuity of mental health presentations. It invites a reconsideration of how psychiatric diagnoses are constructed and applied across the lifespan, emphasizing the importance of distinguishing between neurodevelopmental variation and episodic psychopathology. Importantly, misdiagnosis may have cumulative consequences across the life course, influencing treatment exposure, self-perception, and access to social and occupational opportunities.
